# Age- and Gender-Related Differences in Renal Vascular Responses to Angiotensin II in Rats: The Role of the Mas Receptor

**DOI:** 10.1155/2023/3560468

**Published:** 2023-08-16

**Authors:** Fatemeh Eshraghi-Jazi, Mehdi Nematbakhsh

**Affiliations:** ^1^Water & Electrolytes Research Center Isfahan University of Medical Sciences, Isfahan, Iran; ^2^Department of Physiology, Isfahan University of Medical Sciences, Isfahan, Iran

## Abstract

**Background:**

Renal hemodynamic is influenced by both gender difference and age. Also, the Mas receptor (MasR) as one of the depressor components of the renin-angiotensin system which has more expression in females could postpone some dysfunctions associated with age, although the association between MasR and age in renal vascular responses to angiotensin II (Ang II) in male and female rats was well undefined. Therefore, the current study examined the effects of age and sex on systemic and renal vascular responses to graded doses of Ang II in Wistar rats with or without MasR antagonists (A779).

**Materials and Methods:**

Anesthetized Wistar male and female rats with two age ranges of 8–12 and 24–28 weeks were exposed to cannulate venous and arterial vessels. After stability, mean arterial pressure (MAP), renal perfusion pressure (RPP), renal vascular resistance (RVR), and renal blood flow (RBF) were measured in response to the infusion of Ang II with or without A779.

**Results:**

There were no significant differences in the base values of MAP, RPP, RBF, and RVR between the two genders in both the age ranges of 8–12 and 24–28 weeks. In addition, no significant gender difference was observed in the age ranges of the above mentioned parameters among the groups receiving vehicle or A779. Also, the infusion of vehicle or A779 could not significantly change the base values. On the other hand, the responses of RBF and RVR to Ang II revealed gender differences among 8–12-week groups (*P* < 0.05) but not in 24–28-week groups, while the blockade of MasR could not influence the responses in the age ranges.

**Conclusion:**

It was concluded that age could impress sex difference in RBF and RVR responses to Ang II infusion and that MasR alone could not participate in these responses. In other words, MasR is not active under normal and acutely elevated Ang II levels.

## 1. Introduction

Age is an important factor that affects kidney hemodynamics as well as the renin-angiotensin system (RAS) activity. Both kidneys and RAS participate in the regulation of blood pressure, and advancing age could predispose the prevalence of cardiovascular and kidney diseases in elderly individuals [1]. RAS has two vasoconstrictor and vasodilator arms including angiotensin-converting enzyme (ACE)/angiotensin II (Ang II)/Ang II receptor 1 (AT1R) and ACE2/angiotensin 1–7 (Ang 1–7)/Mas receptor (MasR) axes, respectively [2, 3]. Advancing age increases and reduces the expression of vasoconstrictor and vasodilator arms, respectively [4]. Besides kidney hemodynamics and RAS, gender differences could be overshadowed by age. It was documented that premenopause women have a lower blood pressure than age-matched men, while the gender difference is eliminated by aging [5]. In addition, the serum activity of RAS enzymes such as ACE and ACE2 is variable with respect to age and gender [6].

Several studies have exhibited the role of MasR in systemic and renal vascular responses to Ang II infusion in hypertensive and partial renal ischemia-reperfusion models [7–9]. Considering that MasR may participate to postpone endothelial dysfunction induced by aging [10], the role of MasR in gender- and age-related vascular responses to Ang II in normotensive rats still was not clearly defined. Therefore, the present study investigated the effects of age and gender in systemic and renal vascular responses to the administration of graded doses of Ang II in Wistar rats with or without MasR antagonist (A779).

## 2. Materials and Methods

### 2.1. Animals

Wistar male and female rats used in this study were born and grown in the animal room of the Water and Electrolytes Research Center with a temperature of 23–25°C and a 12 h light/12 h dark cycle. The birth date of each animal was recorded. After weaning, the offspring were classified randomly into two age categories of 8–12 and 24–28 weeks. The animals were housed with free access to water and food until experiment day. The experimental procedures were approved by the Ethics Committee of Isfahan Medical Sciences University (Code # IR. MUI.RESEARCH. REC. 1399.564).

### 2.2. Agents

Drugs used in the current study included urethane (Sigma, USA), A779 (Sigma, USA) as an antagonist of MasR, Ang II (Sigma, USA), heparin sodium ampoule 5000 IU/ml (Darou Pakhsh, Iran), and saline solution 0.9% (Darou Pakhsh, Iran) as a vehicle. The doses of drugs used in the current study (urethane, A779, and Ang II) were determined according to the previous studies [7, 9, 11].

## 3. Experimental Groups

The animals with two age ranges of 8–12 weeks (*n* = 22: 10 males and 12 females) and 24–28 weeks (*n* = 24: 12 males and 12 females) were divided randomly into the following eight experimental groups:

Groups 1 and 2: 8–12-week male (group 1; *n* = 5) and female (group 2; *n* = 6) rats received vehicle before (30 min before Ang II injection) and along with Ang II administration.

Groups 3 and 4: 8–12-week male (group 3; *n* = 5) and female (group 4; *n* = 6) rats received A779 before (30 min before Ang II injection) and along with Ang II administration.

Groups 5 and 6: 24–28-week male (group 5; *n* = 6) and female (group 6; *n* = 6) rats received treatment similar to that in groups 1 and 2, respectively.

Groups 7 and 8: 24–28-week male (group 7; *n* = 6) and female (group 8; *n* = 6) rats received treatment similar to that in groups 3 and 4, respectively.

### 3.1. Surgical Procedure

The animals were anesthetized by urethane (1.7 g/kg; intraperitoneally). After tracheostomy, the carotid and femoral arteries were cannulated by using polyethylene tubes (PE9658 for the carotid artery and PE8040 for the femoral artery, Microtube Extrusions; Australia) filled with saline-heparin solution to measure the mean arterial pressure (MAP) and renal perfusion pressure (RPP), respectively. In addition, to infuse Ang II and A779 (or vehicle), a polyethylene tube filled with saline solution was implanted into the jugular vein. After placing the animal on the right side, an incision was made on the left flank by using an electrosurgical instrument (Erbe Elektromedizin GmbH; Germany), and the left kidney was exposed and warily separated from the surrounding adipose tissues with special care. Then, the left renal artery was carefully isolated, and a flow probe (Transonic Systems Inc.; USA) connected to a perivascular flowmeter system (TS420, Transonic System Inc; USA) was placed around it for measuring renal blood flow (RBF). In order to control RPP during Ang II infusion, an adjustable occluder was fixed around the abdominal aorta just before the branching of the renal artery. Finally, carotid and femoral cannulas connected to the pressure transducers were attached to the power lab system (ADInstruments; Australia). To provide oxygen and ventilation during the experiment, an oxygen tank was connected to the tube implanted into the trachea to use oxygen as needed.

### 3.2. Experimental Design

At first, MAP, RPP, and RBF were measured continuously for at least 30 minutes to stabilize the conditions, and the recording of the last five minutes was considered basal data. Then, groups 3, 4, 7, and 8 received a blouse dose of A779 (50 *μ*g/kg) followed by a continuous dose infusion of A779 (50 *μ*g/kg/h) via a microsyringe pump (New Era Pump Systems, Inc; USA). Also, groups 1, 2, 5, and 6 received vehicle instead of A779. The data of last 3-5 minutes were assigned for evaluating antagonist (or vehicle) effect. After that, all animals received Ang II at doses of 30, 100, 300, and 1000 ng/kg/min using a microsyringe pump without discontinuing A779 (or vehicle) infusion. Each dose was infused for 15 minutes. The recording of the last 3–5 minutes for each dose was considered to evaluate MAP, RPP, and RBF. After ending the experiment, the animals were sacrificed humanely, and the kidneys were immediately removed and weighed. Renal vascular resistance (RVR) was calculated by the RPP/RBF ratio.

### 3.3. Statistical Analysis

Data were analyzed by using SPSS software, version 25 and expressed as the mean ± standard error of the mean (SEM). The analysis of one-way ANOVA followed by the LSD posttest was used to analyze the values of body weight (BW) as well as the weight of the right and left kidneys among groups in both the two age categories. The kidney weight (KW) was represented as both values of raw and normalized based on 100 g BW (KW/100 g BW). The baseline data of MAP, RPP, RBF, and RVR were analyzed by the independent *t*-test. In addition, RBF and RVR responses to A779 or vehicle were reported as the percentage of change from the basal data. Also, RBF and RVR responses to Ang II were reported as the percentage of change from the values after A779 or vehicle infusion, before Ang II infusion. Moreover, repeated measure analysis followed by the LSD posttest was used to analyze all hemodynamic parameters in both stages of A779 (or vehicle) and response to Ang II. The values of *P* < 0.05 were considered significant.

## 4. Results

### 4.1. BW, KW, and KW/100 g BW Measurements

The values of BW, KW, and KW/100 g BW are shown in Table 1. There were significant differences in BW among groups in both the 8–12- and 24–28-week categories (*P* < 0.05). In addition, the values of left and right KWs demonstrated significant changes among the 8–12-week groups (*P* < 0.05). The comparison of the values of left and right KWs found significant differences among the 24–28-week groups (*P* < 0.05), although, no significant difference was observed in the values of the right and left KW/100 g BW (Table 1).

### 4.2. Baseline Measurements

**Figure 1:**
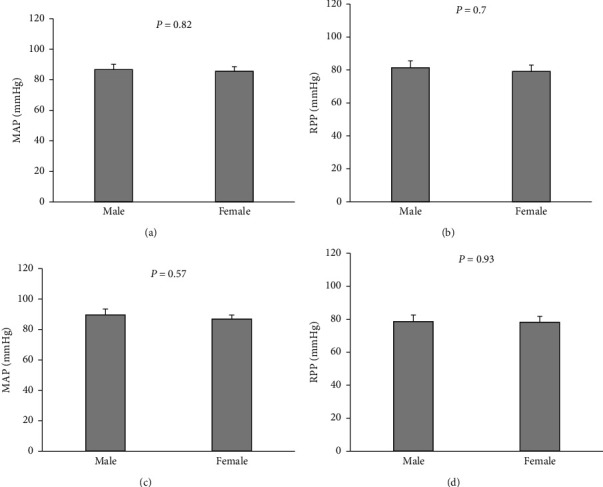
Baseline hemodynamic data of the mean arterial pressure (MAP) and renal perfusion pressure (RPP) in 8–12-week (a, b) and 24–28-week (c, d) animals in both genders. Data were expressed as the mean ± standard error of the mean. There was no significant difference between males and females in both age categories.

**Figure 2:**
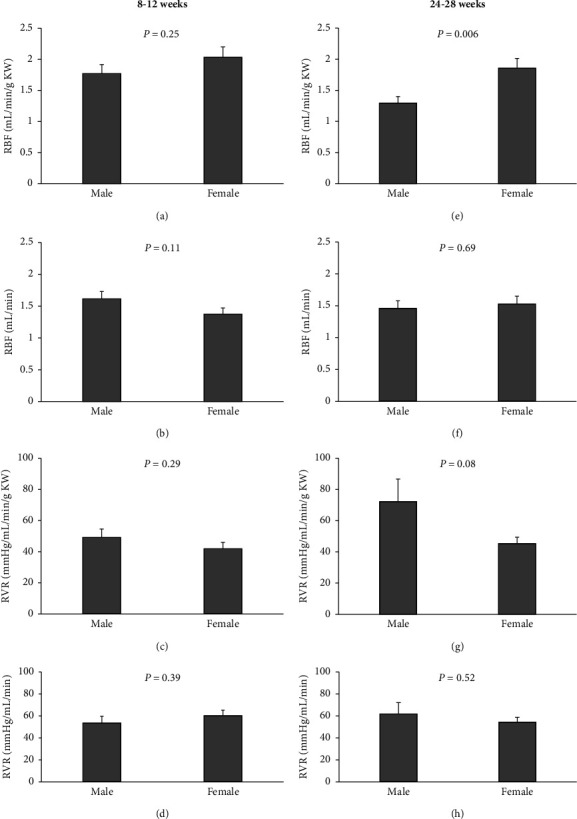
Baseline hemodynamic data of renal blood flow (RBF) and renal vascular resistance (RVR) in 8–12-week (a–d) and 24–28-week (e–h) animals in both genders. Data were expressed as the mean ± standard error of the mean. There was a significant difference in RBF between 24–28-week males and females (*P* < 0.05) (e). The values of RBF and RVR were expressed based on per gram of left wet kidney weight.

MAP, RPP, RBF, and RVR were measured in 10 males and 12 females of 8–12-week rats (Figures 1 and 2). Also, similar parameters were measured in 12 females and 12 males of 24–28-week rats (Figures 1 and 2). There were no significant changes in MAP and RPP between female and male groups in both the age ranges (Figure 1). The values of RBF and RVR either absolute or normalized by KW showed no significant changes between the 8–12-week male and female rats (Figure 2), but there was a significant difference in RBF/KW in the 24–28-week groups (*P* < 0.05) (Figure 2(e)).

### 4.3. Antagonist (A779) Effect Measurements

**Figure 3:**
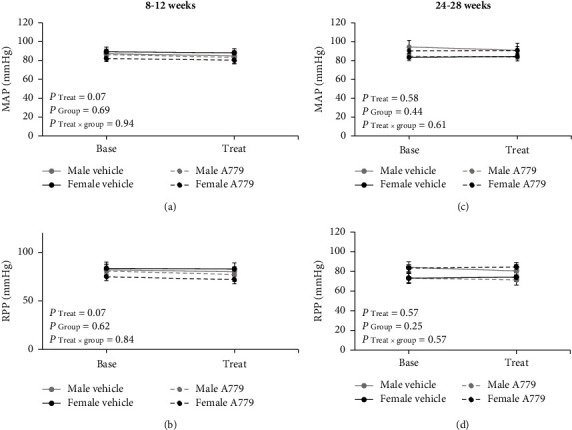
Hemodynamic data of the mean arterial pressure (MAP) and renal perfusion pressure (RPP) before (base) and after (treat) vehicle or A779 infusion in 8–12-week (a, b) and 24–28-week (c, d) animals in both genders. Data were expressed as the mean ± standard error of the mean. There were no significant differences between age-matched males and females receiving vehicle or A779.

**Figure 4:**
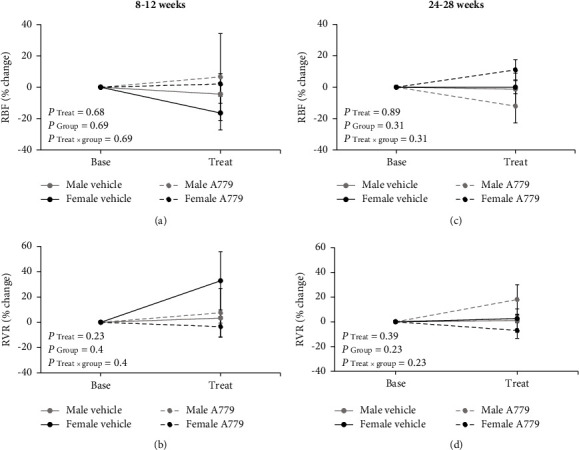
Hemodynamic data of the percentage (%) changes of renal blood flow (RBF) and renal vascular resistance (RVR) before (base) and after (treat) vehicle or A779 infusion in 8–12-week (a, b) and 24–28-week (c, d) animals in both genders. Data were expressed as the mean ± standard error of the mean. There were no significant differences between age-matched males and females receiving vehicle or A779.

The findings showed that the infusion of A779 or vehicle had no significant effect on MAP, RPP, RBF, and RVR in all groups, regardless of age and sex (Figures 3 and 4). Also, no significant differences in MAP and RPP were observed between 8–12-week males and females receiving either vehicle or A779 (Figure 3). Similar observations were seen among the 24–28-week groups (Figure 3). Moreover, the percentage change of RBF and RVR did not exhibit any significant changes between the two genders, regardless of age and treatment (Figure 4).

### 4.4. Vascular Response to Ang II Administration

**Figure 5:**
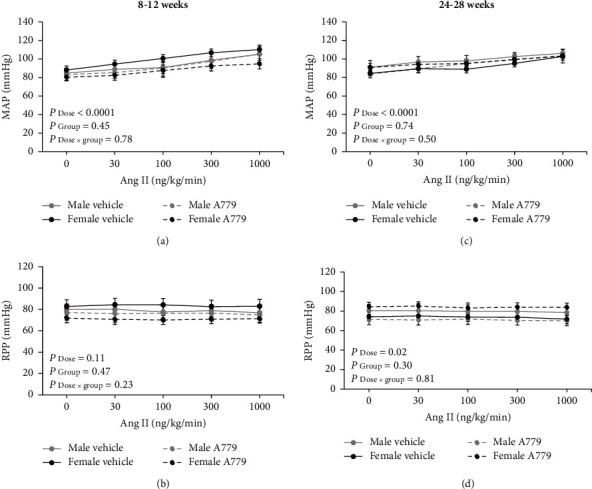
The effect of the vehicle or A779 infusion on the mean arterial pressure (MAP) and renal perfusion pressure (RPP) in response to the infusion of angiotensin II (Ang II) graded doses in 8–12-week (a, b) and 24–28-week (c, d) animals in both genders. Data were expressed as the mean ± standard error of the mean. There was no significant difference between age-matched males and females receiving vehicle or A779.

**Figure 6:**
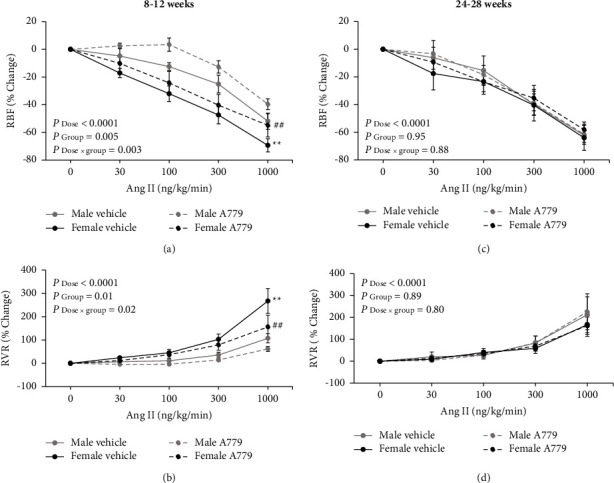
The effect of the vehicle or A779 infusion on the percentage (%) changes of renal blood flow (RBF) and renal vascular resistance (RVR) in response to the infusion of angiotensin II (Ang II) graded doses in 8–12-week (a, b) and 24–28-week (c, d) animals in both genders. Data were expressed as the mean ± standard error of the mean. The symbols ^∗∗^ indicate a significant difference in comparison to the 8–12-week male group receiving vehicle (*P* group ≤ 0.01); the symbols ^##^ indicate a significant difference in comparison to the 8–12-week male group receiving A779 (*P* group ≤ 0.01).

The administration of Ang II graded doses increased MAP in 8–12-week groups receiving either vehicle or A779 (*P*_dose_ < 0.05) (Figure 5(a)). In addition, a similar finding was observed in all 24–28-week groups (*P*_dose_ < 0.05) (Figure 5(c)). The administration of vehicle or A779 induced no significant changes in MAP among the groups in both the age ranges (Figures 5(a) and 5(c)). Also, there were no significant changes in RPP values during the infusion of Ang II graded doses in all 8–12-week groups (Figure 5(b)). Despite adjusting the abdominal aorta diameter by using the occluder during Ang II infusion, RPP values had significant changes in all 24–28-week groups (*P*_dose_ < 0.05) (Figure 5(d)). There were no significant differences in RPP among all groups in both the age ranges (Figures 5(b) and 5(d)). On the other hand, all experimental groups revealed significant changes in RBF and RVR responses to Ang II administration (*P*_dose_ < 0.05, Figure 6). In addition, a significant difference was observed in the percentage change of RBF in response to Ang II between 8–12-week male and female groups receiving either vehicle or A779 (*P*_group_ < 0.05) (Figure 6(a)). In other words, 8–12-week females exhibited more decrement in RBF percentage change than 8–12-week males (Figure 6(a)), and the gender difference was eliminated by increasing age (Figure 6(c)). Moreover, 8–12-week females exhibited more increment in RVR percentage change in response to Ang II than 8–12-week males with or without receiving A779 (*P*_group_ < 0.05) (Figure 6(b)), and the increasing age removed the sex difference among the groups (Figure 6(d)).

## 5. Discussion

The current study's aim was to determine whether the renal hemodynamic responses to the infusion of Ang II with or without receiving A779 were influenced by age and gender. The findings were organized and discussed in some issues. First, the present study exhibited the increment of MAP and RVR as well as the decrement of RBF in response to the infusion of Ang II in all groups receiving vehicle or A779. The literature has documented the dose-dependent pressor responses to Ang II [12–14]. Ang II, as one of the most important peptides of RAS, elicits vasoconstrictor effects mediated by AT1R [2]. Second, the present study revealed that the infusion of Ang II with or without A779 significantly induced a gender difference in the responses of RVR and RBF in the age range of 8–12 weeks with more intensity in female gender. In this regard, some studies examined the AT1aR participation of vascular smooth muscle cells (VSMCs) in renal vascular responses to Ang II in female [15] and male [16] mice. Wolf et al. found that the removal of VSMC-AT1aR in female mice was accompanied with a decrement of 50–75% in vasoconstrictive responses to Ang II [15], and another study reported that the elimination of VSMC-AT1aR in male mice induced a decrease of 20% in vasoconstrictive responses to Ang II [16]. Therefore, the current study hypothesizes that female gender has prominent VSMC-AT1aR in acute peripheral vasoconstrictive responses and suggests further investigations in this regard. In addition, a clinical study reported that normotensive healthy young women had a greater decrement and increment in GFR and RVR in response to Ang II than age-matched men, respectively. The responses exhibited that both gender and AT1R gene polymorphism could determine the changes involved in glomerular filtration, whereas MAP values in response to Ang II were equal in both genders [17]. On the other hand, there were some pieces of evidence against the findings of the current study. One study performed on 8–12-week mice receiving a normal sodium diet indicated the greater responses of MAP and RVR to Ang II in males than in females but without gender difference in RBF response to Ang II, as well as in the expression of AT1R and AT2R and eNOS in the kidney vessels [18]. This contrast is probably due to the lower doses of Ang II in the current study.

Third, the current study found no gender difference in vascular responses to Ang II in the age range of 24–28 weeks. It seems that renal vascular responses to Ang II increased with age in the male sex. Evidence showed that aging induced higher contractile responses to Ang II in males than in females which were paralleled with decreasing the expression of AT2R, while there were not any changes in ACE and AT1R [19]. Aging has destructive effects on the endothelial function. Costa et al. exhibited that the inhibition of NOS decreased contractile responses to Ang II in aged male mice than in aged female mice, whereas the inhibition of NOS increased contractile responses to Ang II in young male and female mice [19]. Also, a decrease in the eNOS expression was observed in the aorta of old male mice [19]. Endothelin also, as a vasoconstrictor agent investigated in gender difference-related subjects [20], could be proposed in renal vascular responses to Ang II with age, especially in the male gender. One study reported that endothelin vasoconstrictor tone is influenced by increasing age in men [21], and therefore, the subject could be known as a risk factor for the prevalence of cardiovascular disease in men with age. Fourth, concerning the role of MasR, the present study achieved an interesting finding. The presence or absence of A779 as a MasR antagonist had no significant effect on vascular responses to Ang II, regardless of age and sex. It was expected that the blockade of MasR intensified the vasoconstrictive effects of Ang II. However, one study reported that the blockade of MasR alone could not influence the vascular response to Ang II in 16-week male and female rats, but the simultaneous blockade of MasR and AT2R attenuated the reduction in vascular response in females only [13]. Therefore, the present study hypothesized that MasR alone was not able to impress vascular responses to Ang II in male and female rats in both 8–12- and 24–28-week age ranges. However, the interaction of MasR and other receptors, especially AT2R, must be examined in future studies. Moreover, the current study suggests that MasR is not active under normal and acutely elevated Ang II levels. It seems that MasR regulates renal hemodynamics only under more chronic conditions such as pregnancy, hypertension, or diabetes. In this regard, one study evidenced that the blockade of MasR alters the RBF and RVR responses to Ang II after moderate renal ischemia reperfusion in female rats, but not in male rats [8]. Another study also reported that the blockade of MasR intensified renal vascular responses to Ang II after partial renal ischemia reperfusion in hypertensive rats [11]. In addition, in spite of decreasing ACE2/Ang 1–7/MasR axis expression with age [4], evidence reported that double deletion of MasR and AT2R, but not single deletion of MasR and AT2R, could participate in the appearance of consequences induced by aging [22]. Therefore, it seems that both MasR and AT2R could take part in attenuating consequences induced by aging. Overall, the present study suggests that the interaction between receptors may involve in the vascular responses to vasoactive agents in various age ranges, and it is recommended that more investigations should be conducted.

### 5.1. Limitation

There is a limitation in this study that could be addressed in future research studies. This study used 8–12- and 24–28-week animals. It is recommended that future studies should use animals with greater age intervals.

## 6. Conclusion

The current study concluded that age could impress sex difference in RBF and RVR responses to Ang II infusion and that MasR alone could not participate in these responses. In other words, MasR is not active under normal and acutely elevated Ang II levels. It is suggested to evaluate the effects of dual blockade of MasR and another receptor, especially AT2R.

## Figures and Tables

**Table 1:** The values of body weight (BW), kidney weight (KW), and KW/100 g BW in all experimental groups.

Age	Gender	Group	BW (g)	Left KW (g)	Left KW (g)/100 g BW	Right KW (g)	Right KW (g)/100 g BW
8–12 weeks	M	Vehicle	242.6 ± 16.91	1 ± 0.06	0.41 ± 0.01	1 ± 0.06	0.41 ± 0.01
	F	Vehicle	164.5 ± 7.17^*∗∗∗*^	0.67 ± 0.02^*∗∗∗*^	0.41 ± 0.02	0.67 ± 0.02	0.41 ± 0.02
	M	A779	221.8 ± 8.16	0.84 ± 0.04^*∗*^	0.38 ± 0.01	0.84 ± 0.04^*∗*^	0.38 ± 0.02
	F	A779	171.66 ± 5.32^**##**^	0.7 ± 0.03^**#**^	0.40 ± 0.01	0.69 ± 0.03^**#**^	0.4 ± 0.01
	*P* value		0.0001	0.0001	0.52	0.0001	0.58

Age	Gender	Group	BW (g)	Left KW (g)	Left KW (g)/100 g BW	Right KW (g)	Right KW (g)/100 g BW

24–28 weeks	M	Vehicle	319.66 ± 13.72	1.15 ± 0.05	0.36 ± 0.01	1.1 ± 0.05	0.34 ± 0.01
	F	Vehicle	211.5 ± 6.87^**∆∆∆**^	0.77 ± 0.01 ^**∆∆∆**^	0.36 ± 0.01	0.75 ± 0.02^**∆∆∆**^	0.35 ± 0.01
	M	A779	334.16 ± 17	1.1 2 ± 0.04	0.33 ± 0.01	1.07 ± 0.05	0.32 ± 0.01
	F	A779	227.5 ± 7.57^**$$$**^	0.87 ± 0.03^**$$$**^	0.38 ± 0.01	0.85 ± 0.03^**$$**^	0.37 ± 0.02
	*P* value		0.0001	0.0001	0.1	0.0001	0.28

Data were expressed as the mean ± standard error of the mean. ^*∗*^*P* < 0.05 and ^*∗∗∗*^*P* < 0.001 represent significant differences in comparison to the 8–12-week male receiving vehicle; ^#^*P* < 0.05 and ^##^*P* < 0.01 show significant differences in comparison to the 8–12-week male receiving A779; ^∆∆∆^*P* < 0.001 shows a significant change compared with the 24–28-week male receiving vehicle; ^$$^*P* < 0.01 and ^$$$^*P* < 0.001 reveal significant differences in comparison to the 24–28-week male receiving A779.

## Data Availability

The data used to support the findings of the study are available from the corresponding author upon request.
